# Supplementary material for: Lower Concentrations of Amphotericin B Combined with Ent-Hardwickiic Acid Are Effective against Candida Strains
Source: Antibiotics (Basel). 2023 Mar 3;12(3):509. doi: 10.3390/antibiotics12030509 (PMC10044661; doi:10.3390/antibiotics12030509)
Supplement: Supplementary file 1 [file antibiotics-12-00509-s001.zip › antibiotics-2161977-supplementary.pdf]

antibiotics

MDPI

*Supplementary*

# Lower Concentrations of Amphotericin B Combined with *Ent*-Hardwickiic Acid Are Effective against *Candida* Strains

**Maria V. Sousa Teixeira, Jennyfer A. Aldana-Mejía, Márcia E. da Silva Ferreira and Niege A. J. Cardoso Furtado ***

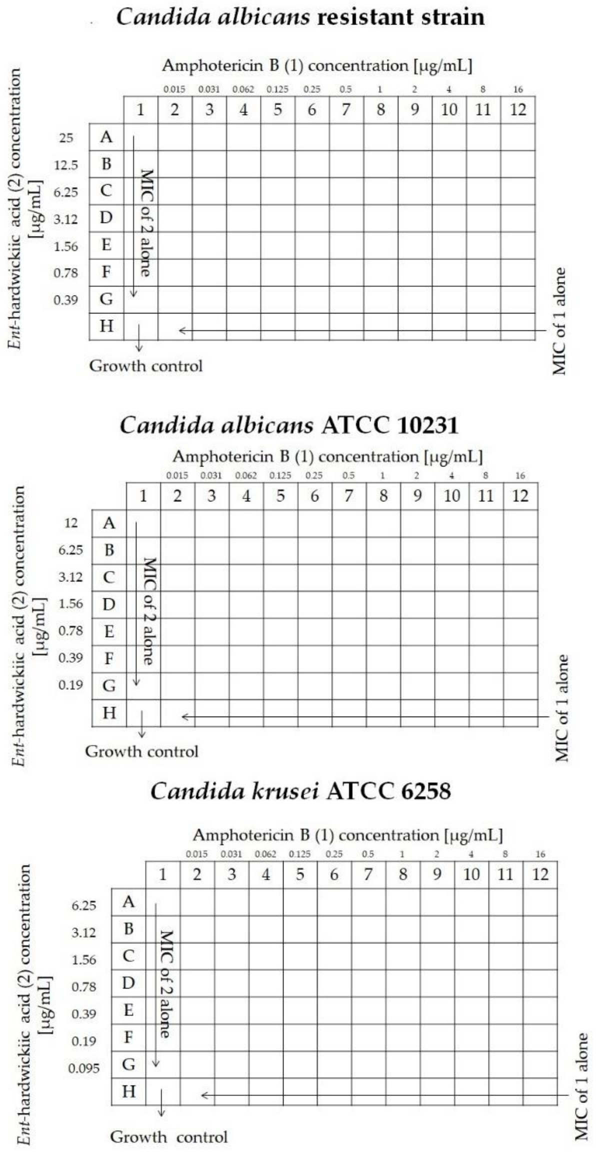


**Figure S1.** Combinations of amphotericin B (1) and *ent*-hardwickiic acid (2) concentrations (µg/ mL) against *Candida* strains in the 96 well microplates used in the checkerboard microdilution method.
